# How do socioeconomic determinants of health affect the likelihood of living with HTLV-1 globally? A systematic review with meta-analysis

**DOI:** 10.3389/fpubh.2024.1298308

**Published:** 2024-01-24

**Authors:** Nydile Ramesh, Beatrice Cockbain, Graham P. Taylor, Carolina Rosadas

**Affiliations:** ^1^School of Public Health, Imperial College London, London, United Kingdom; ^2^Section of Virology, Department of Infectious Disease, Faculty of Medicine, Imperial College London, London, United Kingdom; ^3^National Centre for Human Retrovirology, St Mary’s Hospital, Imperial College Healthcare NHS Trust, London, United Kingdom

**Keywords:** HTLV-1, education, income, employment, social determinant of health

## Abstract

**Introduction:**

Human T Lymphotropic Virus type 1 (HTLV-1) is a neglected retrovirus associated with many clinical disorders, most notably Adult T-cell Leukemia/Lymphoma and HTLV-1-Associated Myelopathy (HAM). Found in endemic clusters across the world, high prevalence has been reported in minoritized groups who suffer from health inequities. This study investigates the association between HTLV-1 prevalence and the following socioeconomic determinants of health: education, income, and employment, which are markers of health inequity.

**Methods:**

A systematic review was conducted by searching the following databases: Ovid/Medline, Embase, Global Health Database, Web of Science, LILACS and SciELO. Primary studies in English, Spanish and Portuguese mentioning HTLV-1 and one of education, income and/or employment were included. A random-effects meta-analysis was performed, and odds ratios (OR) were calculated to determine the association between these socioeconomic determinants of health and HTLV-1 prevalence.

**Results:**

42 studies were included. The likelihood of having HTLV-1 was higher in individuals with less than completed primary education compared to those who completed primary education (OR 1.86 [95% CI 1.34–2.57]; *p* < 0.01). This may be because individuals with low education have reduced access to and understanding of health information, thus increasing the prevalence of risk factors associated with HTLV-1 infection. No other determinants were found to be statistically significant.

**Conclusion:**

Fewer years of schooling are associated with increased likelihood of contracting HTLV-1. Therefore, health promotion materials and public health policies regarding HTLV-1 must consider those with lower educational levels to effectively reduce disease transmission.

**Systematic review registration:**

https://www.crd.york.ac.uk/prospero/display_record.php?RecordID=335004, identifier (CRD42022335004).

## Introduction

Human T Lymphotropic virus type 1 (HTLV-1) is a human retrovirus that causes chronic lifelong infection, primarily of T-lymphocytes (1). First discovered in 1980 (2), it has been found in endemic clusters in many regions globally, such as West Africa, Brazil and Japan (3). In 2012 it was estimated that at least 5–10 million people were living with HTLV-1 worldwide (4), however this is thought to be an underestimate since reliable prevalence data is not available for many areas of the world (3). HTLV-1 infection is commonly associated with marginalized groups such as sex workers, immigrants, and First Nations communities (5). Furthermore, routes of transmission are varied, being through contaminated blood products, vertical transmission via prolonged breastfeeding, and condomless sexual intercourse (6).

HTLV-1 infection can cause severe and potentially fatal complications. The virus is associated with a wide variety of clinical disorders, most notably a severe leukemia and lymphoma, Adult T-cell Leukamia/Lymphoma (ATL) and a chronic and progressive neurological disease, known as HTLV-1-associated myelopathy (HAM) (7). Additionally, a meta-analysis revealed that HTLV-1 infection is associated with a 57% increase in all-cause mortality, as well as several inflammatory conditions such as uveitis, infective dermatitis and polyarthritis (5). No curative treatment exists for HTLV-1 and there is a lack of screening for the infection globally (7). Currently, prevention remains the best option to reduce HTLV-1 prevalence internationally.

HTLV-1 infection can impact quality of life significantly and lead to health inequities (8). The World Health Organization (WHO) defines health inequities as “systematic differences in the health status of different population groups. These inequities have significant social and economic costs both to individuals and societies” (9). By definition, health inequities are unjust and may be avoidable with the implementation of adequate health policies (9).

Health inequities are influenced by the social determinants of health, which are defined as “the non-medical factors that influence health outcomes” (10), and can include factors such as education, income and employment. The links between reduced access to such factors and increased prevalence of infectious diseases are well-described, in different populations and infections globally (11). Educational attainment is inversely correlated with the prevalence of several sexually transmitted infections, including HIV, *Treponema pallidum*, *Chlamydia trachomatis*, and *Neisseria gonorrhea* (12–14). Low income is also associated with an increased susceptibility to a range of communicable diseases including HIV, tuberculosis, hepatitis, dengue, pneumonia, cholera, and diarrhoeal diseases (15). Unemployment is associated with a deterioration in physical and mental health (16) and has been seen to confer 2–3 times higher risk mortality from COVID-19 infection (17).

Scattered data indicates that HTLV-1 infection is also linked with many social determinants of health, including employment, income, education, sex work, intravenous drug use (IDU), homelessness, immigration, sexuality, and gender (3, 8, 18). However, to date there has been no systematic assessment of how the prevalence of HTLV-1 infection is affected by socioeconomic determinants of health. Therefore, this study aims to address this gap by exploring the association between HTLV-1 prevalence and three socioeconomic determinants of health: education, income and employment.

## Methods

**Figure 1:**
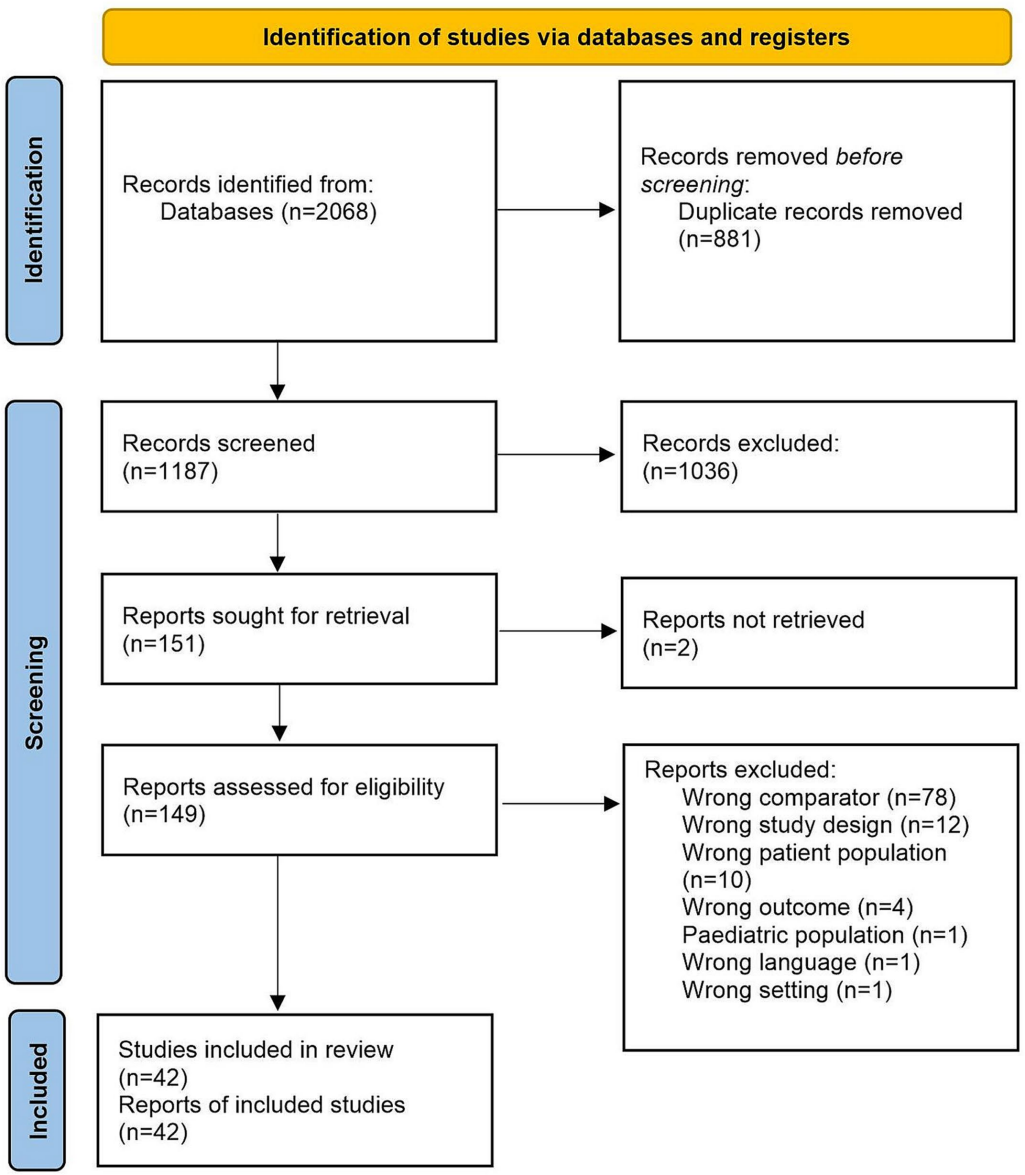
PRISMA diagram. Of the 2068 papers screened, 175 were found by searching Ovid/MEDLINE, 156 were found in Embase, 344 were found in the Global Health database, 552 were found in Web of Science, 27 were found in Latin American and Caribbean Health Sciences Literature (LILACS) and 814 were found in SciELO.

The Preferred Reporting Items for Systematic Reviews and Meta-Analysis (PRISMA) reporting guidelines were followed throughout this study (Figure 1). The review was registered in the International Prospective Register of Systematic Reviews (PROSPERO) database (CRD42022335004) (19). The populations studied included blood donors, pregnant women, and the general population. The odds of having HTLV-1 according to different socioeconomic determinants of health were measured and compared to various control groups. These determinants of health included educational attainment, income, and employment.

### Search strategy

A total of six databases were systematically searched by one reviewer (NR) on 23rd February 2022. These included Ovid/MEDLINE, Embase, The Global Health database, Web of Science, Latin American and Caribbean Health Sciences Literature (LILACS) and SciELO. The search strategy included the following groups of search terms: all key terms referring to HTLV-1, education, income, and employment. All search terms for HTLV-1 were combined with all search terms related to one socioeconomic determinant of health. This process was repeated for all three determinants of health. The search terms and examples of searches are outlined in Supplementary Tables S1, S2.

### Eligibility criteria

#### Inclusion criteria

Studies considered for inclusion were those written in English, Spanish or Portuguese, containing primary research about HTLV-1 prevalence with mention of one or more of the following determinants: education, income or employment. The search was not restricted by date or study design.

#### Exclusion criteria

Duplicate studies, studies about HTLV-2, HIV, and those that did not contain primary research were also excluded.

### Study selection

One reviewer (NR) screened the title and abstracts of all eligible studies using Covidence (Veritas Health Innovation, Melbourne, Australia), a screening tool designed to assist with systematic reviews. Duplicates and studies which did not fit the eligibility criteria were excluded. A second reviewer (BC) screened 10% of the title and abstracts randomly selecting papers using an online random number generator. After the initial screening, one reviewer (NR) screened all remaining papers in full for the final selection, whilst a second reviewer (BC) screened the full texts of 10% of randomly selected papers. The concordance rate between both reviewers at both stages was found to be 99.7 and 98.7% respectively, so no additional duplicate screening was deemed necessary. Any discrepancies were resolved by a third reviewer (CR).

### Statistical analysis

The data were analyzed using RevMan 5.4. The prevalence of HTLV-1 within each population was used to calculate the odds ratios (OR) for dichotomous determinants. Each determinant was analyzed using the random effects Mantel–Haenszel model to determine if the association between HTLV-1 and any determinant was statistically significant. Heterogeneity was also calculated and represented as I^2^.

Education was further divided into three categories: did not complete primary education, did not complete secondary education, and completed secondary education and above. There was overlap between these classifications, therefore separate subgroup analyzes were carried out to understand the isolated effect that each level of education had on HTLV-1 prevalence. Similarly, income was divided into two categories: low and middle/high income, and low/middle and high income to again determine if differing levels of income impacted results.

There was no standardized way to categorize education across all studies, therefore for the purpose of this review, it was classified as the following: primary education 1–8 years, secondary education 9–12 years, higher education 12+ years. This was adapted from school systems in Brazil, where many of the selected studies took place (20). The classification of income and employment were derived from the definitions used in the selected studies (See Supplementary Table S3).

Where data were available, subgroup analysis was conducted for each determinant, according to the populations studied: blood donors, pregnant women, and the general population. Additionally, analysis including studies with only explicit confirmatory testing for HTLV-1 was carried out for all determinants. This had no impact on the findings; therefore, these data are not shown. Forest plots for each socioeconomic determinant of health were created.

Due to the nature of the topic, the existing literature mainly consisted of observational studies. Therefore, no specific quality assessment was undertaken for the papers in this study.

### Research ethics approval

Ethics approval was not required as this was a systematic review with meta-analysis and did not include patient involvement.

### Patient and public involvement

Patients were not involved in the study design, data collection, data analysis, data interpretation or writing of the report.

## Results

The search strategy yielded 2068 papers in total (Figure 1). Of these, 175 were found by searching Ovid/MEDLINE, 156 were found in Embase, 344 were found in The Global Health database, 552 were found in Web of Science, 27 were found in Latin American and Caribbean Health Sciences Literature (LILACS) and 814 were found in SciELO.

After screening, 2022 papers were excluded, and 42 studies were included for meta-analysis (Figure 1). Reasons for exclusion include wrong comparator, wrong patient population or irrelevant data. Data were included from over 500,000 participants in 11 countries. Thirty-six studies (86%) took place in LMICs, with 16 (38%) taking place in Brazil (2, 21–35) and the rest being carried out in Peru (36–38), Guadeloupe (39, 40), Mozambique (41), Nigeria (42, 43), Iran (44–50), Jamaica (51), Gabon (52) and Panama (53) (Supplementary Table S3). Thirty-seven papers assessed level of education in relation to HTLV-1 infection (21–39, 41, 42, 44–51, 54–61). Eighteen papers assessed income (23, 26–30, 33, 35, 39, 40, 43, 47, 48, 50, 51, 53, 56, 62), and six assessed employment status (42, 47, 50, 52, 55, 59). The characteristics of selected studies are summarized in Supplementary Table S3.

Thirty-seven papers assessed level of education in relation to HTLV-1 infection (21–39, 41, 42, 44–51, 54–61). Of these, twelve papers found a statistically significant association between education and the likelihood of having HTLV-1 (21, 31, 34, 36, 39, 45, 48, 50, 54, 57, 58, 60). Eighteen papers assessed income (23, 26–30, 33, 35, 39, 40, 43, 47, 48, 50, 51, 53, 56, 62), and of these, only four papers found a statistically significant association between income and the likelihood of having HTLV-1 (39, 40, 47, 48). Lastly, six papers assessed employment (42, 47, 50, 52, 55, 59) and none found a statistically significant association for this variable. The characteristics of selected studies are summarized in Supplementary Table S3.

**Figure 2:**
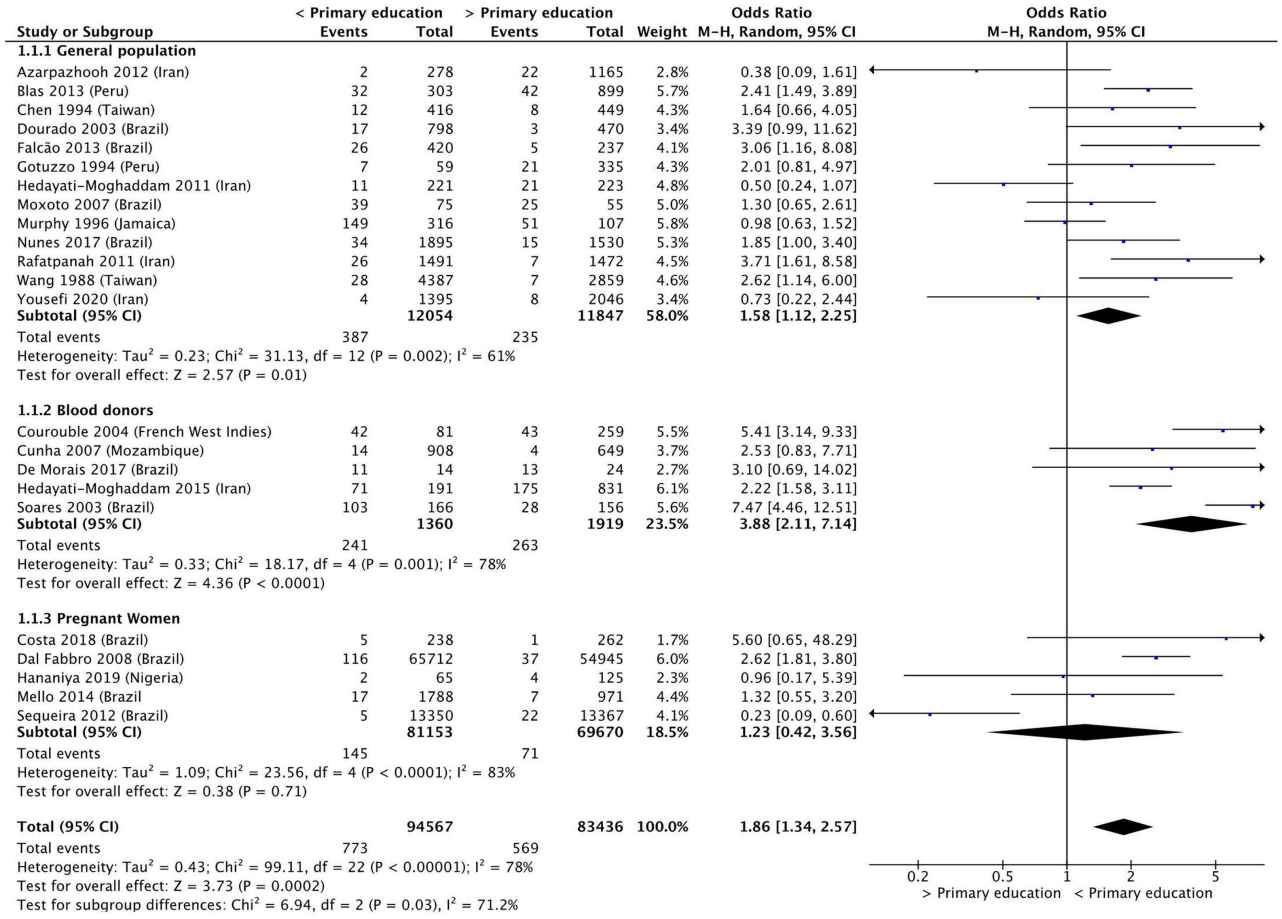
Forest plot comparing the level of primary education and HTLV-1 prevalence, subdivided into the groups of general population, blood donors and pregnant women.

**Figure 3:**
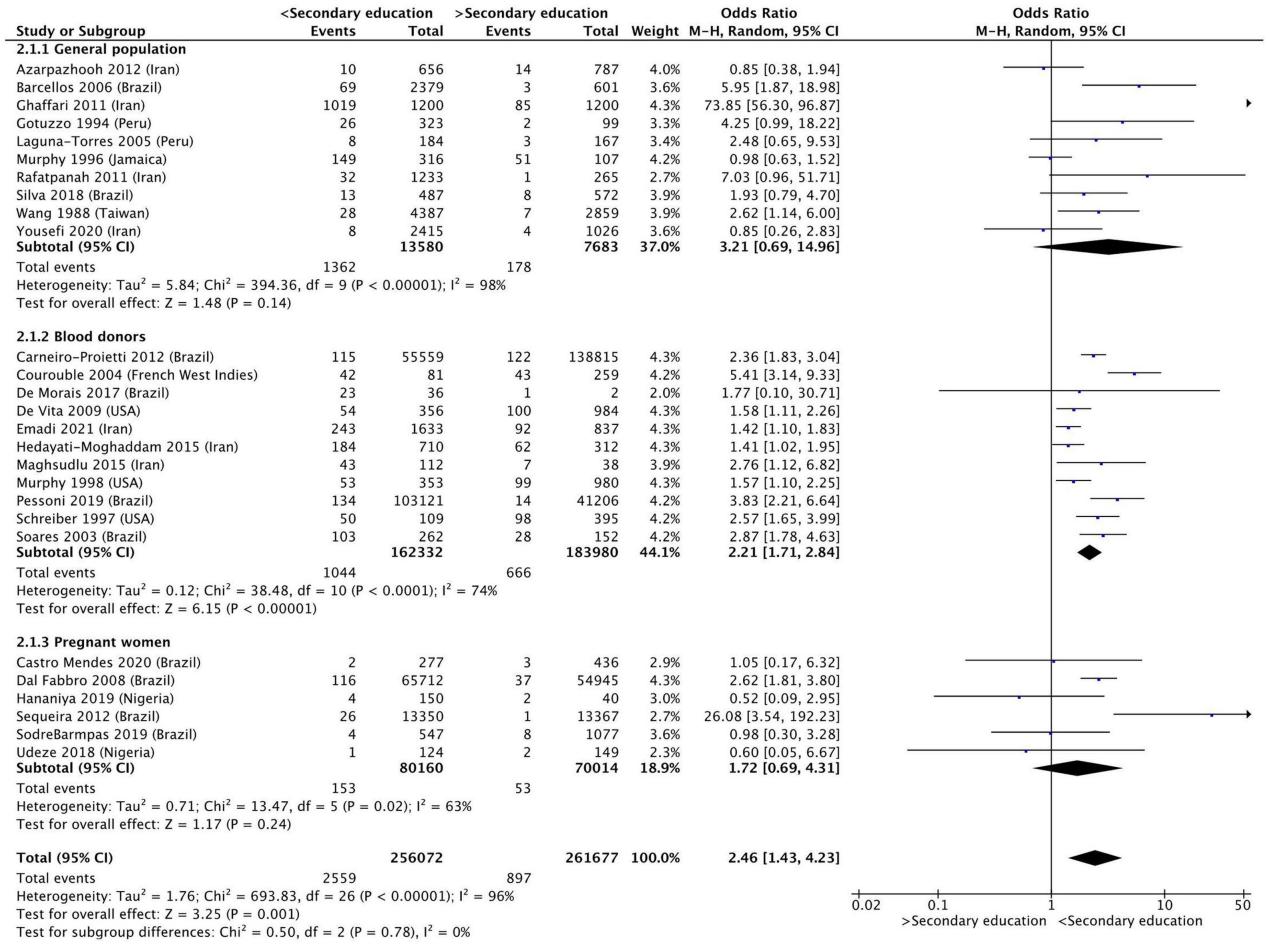
Forest plot comparing the level of secondary education and HTLV-1 prevalence, subdivided into the groups of general population, blood donors and pregnant women.

Individuals with less than primary education were almost twice as likely to be infected with HTLV-1, compared to those who had completed primary education (OR 1.86 [95% Confidence Interval (CI) 1.34–2.57]; *p* < 0.01) (Figure 2) or had completed secondary education (OR 2.46 [95% CI 1.43–4.23]; *p* < 0.01) (Figure 3). Considerable heterogeneity was observed for both variables, with I^2^ = 78% and I^2^ = 96% for primary and secondary education, respectively. To determine whether primary or secondary education was predominantly influencing the results, the prevalence of HTLV-1 among those who had not completed secondary education was compared with those having completed secondary education (Supplementary Figure S1). Having completed secondary education was associated with a significantly lower rate of HTLV-1 infection (OR 0.47 [95% CI 0.35–0.61]; *p* < 0.01, I^2^ = 0%). In a subgroup analysis only the results for blood donors were statistically significant (OR 0.44 [95% CI 0.32–0.59]; *p* < 0.01, I^2^ = 0%) (Supplementary Figure S1). These results suggest that completing secondary education is associated with low odds of being seropositive for HTLV-1.

Subgroup analysis for those who had incomplete and complete primary education found that the results for the general population (OR 1.58 [95% CI 1.12–2.25]; *p* < 0.01, I^2^ = 61%) and blood donors (OR 3.88 [95% CI 2.11–7.14]; *p* < 0.01, I^2^ = 78%) remained statistically significant, whereas that for pregnant women (OR 1.23 [95% CI 0.42–3.56]; *p* = 0.71, I^2^ = 83%) were no longer significant (Figure 2).

For those who had incomplete and complete secondary education, subgroup analysis found that the results for blood donors (OR 2.02 [95% CI 1.58–2.58]; p < 0.01, I^2^ = 74%) remained significant, whereas the results for the general population (OR 4.01 [95% CI 0.89–18.10]; *p* = 0.07, I^2^ = 96%) and pregnant women (OR 1.72 [95% CI 0.69–4.31]; *p* = 0.24, I^2^ = 63%) were no longer significant (Figure 3).

**Figure 4:**
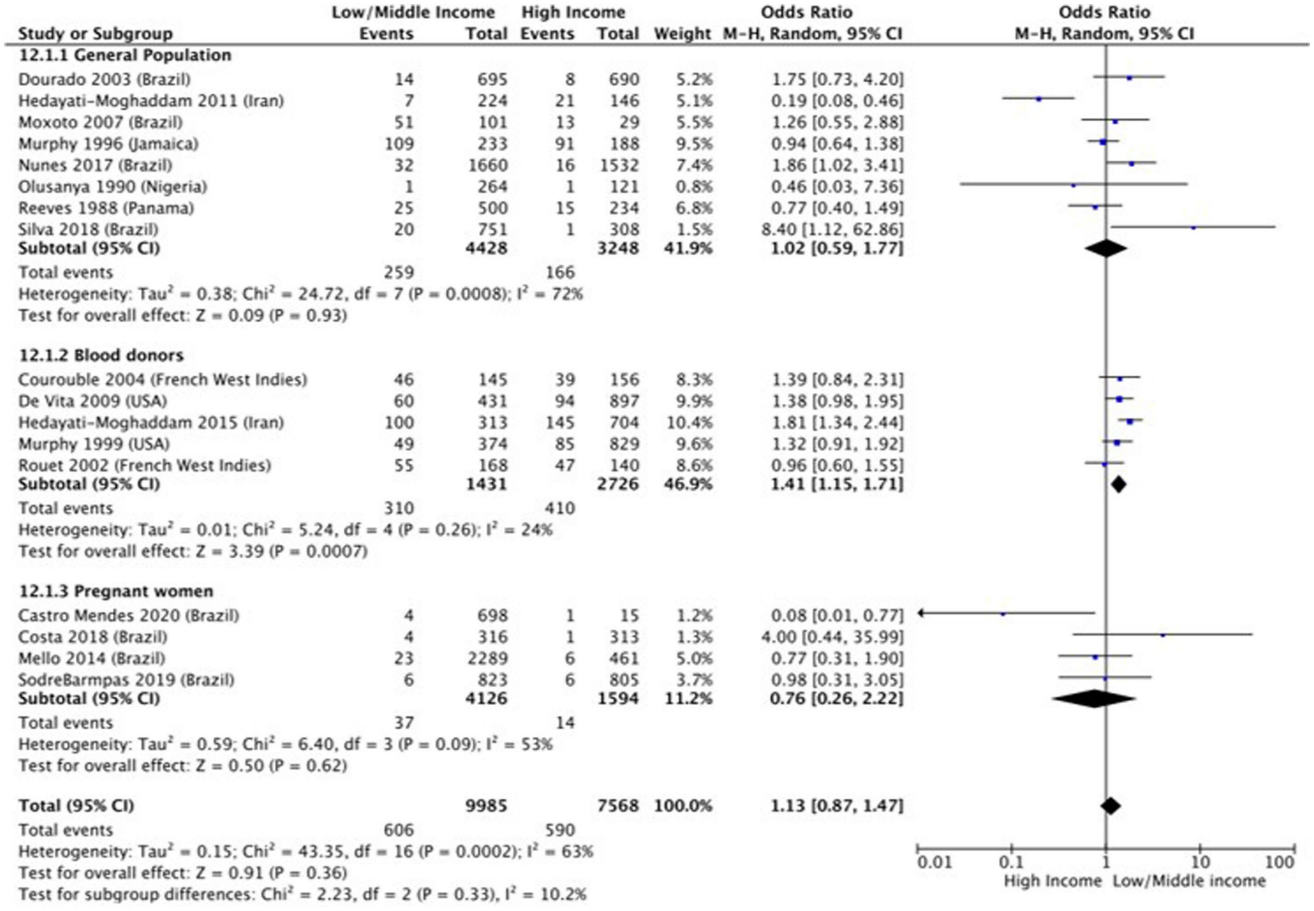
Forest plot comparing low and middle/high income and HTLV-1 prevalence, subdivided into the groups of general population, blood donors and pregnant women.

**Figure 5:**
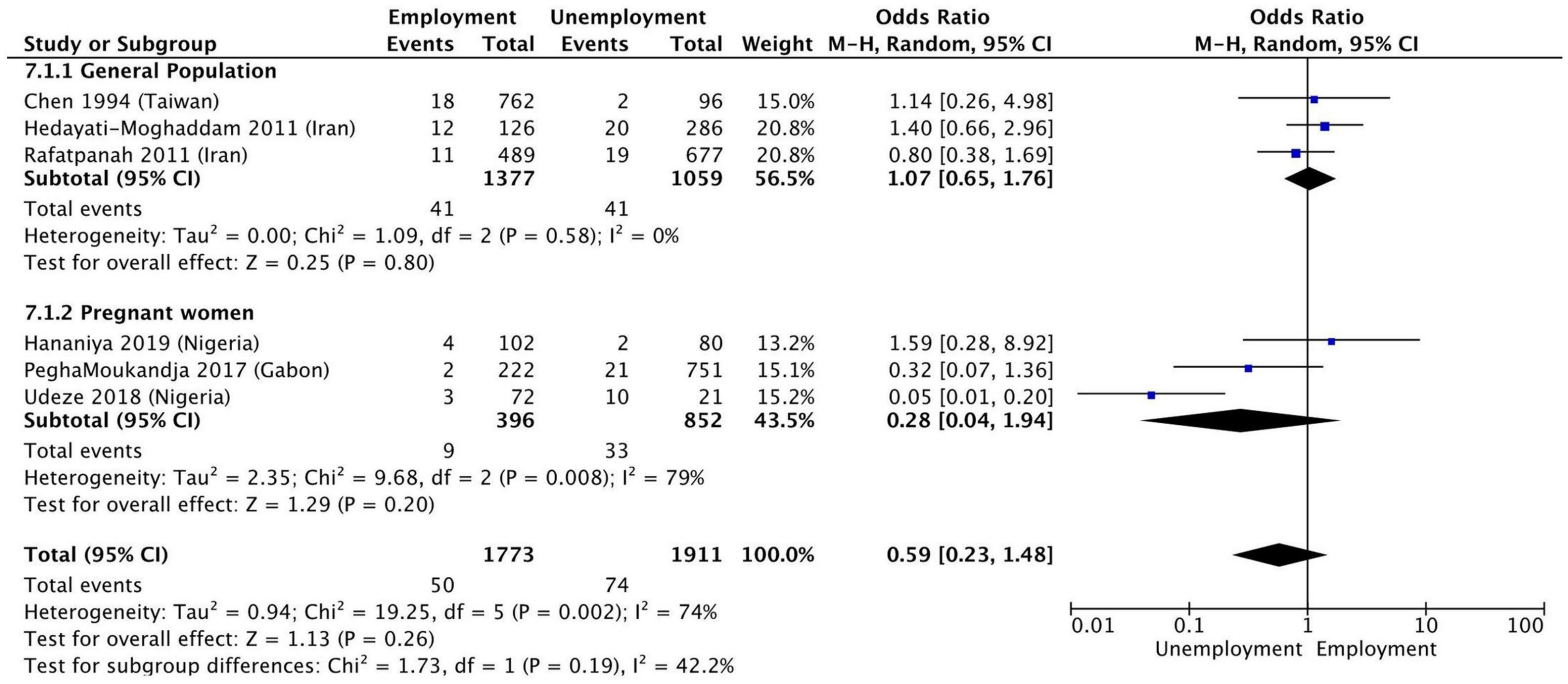
Forest plot comparing employment status and HTLV-1 prevalence, subdivided into the groups of general population, blood donors and pregnant women.

Income (OR 1.1 [95% CI 0.87–1.47]; *p* = 0.36, I^2^ = 65%) (Figure 4) and employment status (OR 0.59 [95% CI 0.23–1.48] *p* = 0.26, I^2^ = 74%) (Figure 5) were not found to be associated with HTLV-1 infection. However, the subgroup analysis for income found that amongst blood donors, low income was associated with increased odds of being infected by HTLV-1 (OR 1.41 [95% CI 1.15–1.71]; *p* < 0.01, I^2^ = 24%) (Figure 4). No other subgroup analysis had an impact on the overall result (Figures 4, 5).

## Discussion

HTLV-1 is a neglected infection that mainly affects vulnerable groups. However, there is little systematic data on the impact of socioeconomic determinants of health on HTLV-1 prevalence globally. Some existing studies touch on the relationship between HTLV-1 prevalence and education, socioeconomic status, social class, age at first intercourse, parity, and race/ethnicity, however, there has been inadequate action by governing bodies in response to these results (63–65).

The present study revealed that lower education is associated with higher likelihood of having HTLV-1 infection. Initially it appeared that having completed secondary education (including those who had some degree of higher education) was associated with reduced prevalence of HTLV-1 infection, but further analysis demonstrated that incomplete primary education, i.e., not completing at least 8 years of education, contributed most to the likelihood of having HTLV-1 infection. This has major implications for effective public health education. No other socioeconomic determinant of health analyzed (income and employment status) was found to be significant overall, except for low income among blood donors in the subgroup analysis.

Level of schooling is an important social determinant of health since less education can lead to social and health inequities. Education level is just one measure of socioeconomic status and is closely linked with various determinants of health, making it difficult to establish the isolated effect on HTLV-1 prevalence. Despite this, several studies have confirmed that fewer years of education are associated with poorer health literacy (66–72). Health literacy is defined as the ability to find, understand, and use health information (73). Reduced access to and understanding of health promotion materials can directly impact an individual’s risk of acquiring HTLV-1 infection (30). Indeed, Nunes et al. (30), found illiteracy to be associated with lower rates of condom use.

Furthermore, fewer employment opportunities are available for individuals with lower levels of education (74). This can restrict the type of work available, potentially worsening health inequities, as well as restricting individuals to types of work which may increase their risk of HTLV-1 transmission, such as condomless sex work (75).

Fewer years of schooling can not only reduce employment opportunities but also associated income. Low income may affect access to healthcare and may lead to individuals being more reliant on financial support from partner(s). This support may be more assured in the context of sexual relationships, particularly condomless sex and childbearing (76, 77). Condomless sex, especially with multiple partners, is a risk factor for acquiring infection, and can increase HTLV-1 prevalence (78). Moreover, low education level and financial dependency are both independently associated with higher rates of violence against women (79), and this in turn has also been linked to a 9-fold increased risk of having HTLV-1 (80). Additionally, it is possible that low parental income may predispose children to low education levels, which our study found is associated with increased risk of HTLV-1 prevalence (81).

Our study found no statistically significant impact of income and employment status on the prevalence of HTLV-1 infection. However, low income was associated with the increased likelihood of blood donors being seropositive for HTLV-1. The lack of overall association was surprising as low income is usually associated with low education level, the determinant of health assessed in this study that was found to be significant. Sampaio et al. (82) recently compared HTLV-1 prevalence and Human Development Index (HDI) of various countries globally and found that HTLV-1 prevalence was three times higher in countries with low HDI than in countries with high HDI (82). HDI is calculated using education level and GDP *per capita*, confirming that these determinants of health influence HTLV-1 prevalence globally. As our study assessed the impact on an individual level, differences in methodologies including the variety of countries that were included in the studies, may have influenced the results.

The results of our study will have been impacted by the limited number of studies available, particularly from countries other than Brazil, the wide period of time that the studies were performed, with some of which being done more than three decades ago, and the heterogeneity observed. Information on education was collated for over 500,000 individuals, representing 84% of the total data which increased the likelihood of detecting an association (See Figure 1). Furthermore, much of the evidence used differing methodologies, and measured variables differently. Additionally, there was limited access to the raw data from each study, making it difficult to standardize the way in which the different determinants of health were classified for this analysis. This contributed greatly towards heterogeneity within the data and limited depth. In addition to this, many studies did not account for confounding factors. Therefore, although some studies have found a significant association between HTLV-1 prevalence and low income (27, 39, 47, 48) and employment status (47), this was not confirmed by this meta-analysis. Thus, further research in this area is needed.

Upcoming studies on HTLV-1 should therefore aim to investigate various factors affecting the prevalence of infection globally, such as immigration and race, and detailed information on socioeconomic determinants of health should be included in every future prevalence study. These results will be important in solidifying our understanding of the topic and may aid both clinicians and policy makers in improving access to HTLV-1 prevention strategies and care.

Since HTLV-1 affects many marginalized populations across the world, an improved understanding of the virus’ epidemiology in diverse populations is necessary, to try and reduce the impact of the infection on these groups. This further reinforces the need for research in other areas of the world with high HTLV-1 prevalence to consider the structural inequities associated with the infection. Our findings emphasize the importance of ensuring that health promotion strategies, including those to reduce HTLV-1 prevalence, are designed to be accessible to those with poor health literacy. For example, this could be achieved by using minimal text on posters (63), via art (83), or video-based campaigns (84).

This concept is hardly new, since there are many examples of successful health promotion strategies that focus on providing information to at-risk populations. In particular, public communication campaigns regarding COVID-19 and national lockdowns were successful in significantly reducing mortality from SARS-CoV-2 in New Zealand (85). With information about the importance of handwashing and staying at home being relayed to the population in an accessible way, a high degree of national support for the intervention emerged, despite the disruption to people’s lives (85). Furthermore, the introduction of school-based sex education in the United Kingdom, in which information was delivered using appropriate language, was found to be effective at reducing the risk of contracting sexually transmitted infections in early adulthood (86). These studies further strengthen our findings and emphasize the importance of accessible health information, especially in relation to HTLV-1.

In conclusion, HTLV-1 is a severely neglected infection in both medical research and practice, despite affecting millions of people worldwide and having a significant impact on their quality of life. It is associated with marginalized populations, who disproportionally suffer from the consequences of chronic HTLV-1 infection due to the wider consequences of living with health inequities. This study found that individuals with less primary education were more likely to have HTLV-1 infection, compared to those with higher levels of education. This highlights the importance of ensuring health promotion materials regarding HTLV-1 are accessible to people of all education levels. This will be helpful to reduce HTLV-1 transmission and the significant morbidity and mortality associated with the infection.

## Data availability statement

The original contributions presented in the study are included in the article/Supplementary material, further inquiries can be directed to the corresponding author.

## Author contributions

NR: Conceptualization, Data curation, Formal analysis, Methodology, Writing – original draft. BC: Conceptualization, Data curation, Methodology, Supervision, Writing – review & editing. GT: Conceptualization, Supervision, Writing – review & editing. CR: Conceptualization, Methodology, Supervision, Writing – review & editing.
