# Supplementary material for: How do socioeconomic determinants of health affect the likelihood of living with HTLV-1 globally? A systematic review with meta-analysis
Source: Front Public Health. 2024 Jan 24;12:1298308. doi: 10.3389/fpubh.2024.1298308 (PMC10848500; doi:10.3389/fpubh.2024.1298308)
Supplement: Supplementary file 1 [file Table_1.docx]

*Table S1: Search terms used in this systematic review*

| **Table of search terms** | |
| --- | --- |
| HTLV-1 | Health inequities |
| HTLV-1  HTLV-1 Infections  Human T-Lymphotropic Virus 1  Human T-cell Lymphotropic Virus 1  Human T cell Leukaemia Virus 1 | Education  Education  Educational attainment  Educational status  School  Schooling  Income  Income  Salaries  Benefits  Earning  Minimum wage  Wage  Socioeconomic factors  Socioeconomic status  Social class  Employment  Employment  Unemployment  Poverty |
