# Supplementary material for: How do socioeconomic determinants of health affect the likelihood of living with HTLV-1 globally? A systematic review with meta-analysis
Source: Front Public Health. 2024 Jan 24;12:1298308. doi: 10.3389/fpubh.2024.1298308 (PMC10848500; doi:10.3389/fpubh.2024.1298308)
Supplement: Supplementary file 2 [file Table_2.docx]

*Table S2: Examples of searches used in this systematic review*

| Determinant of health | Examples of search terms |
| --- | --- |
| Education | ("HTLV-1" OR "HTLV-1 Infections" OR  "Human T-Lymphotropic Virus 1" OR  "Human T cell Lymphotropic Virus 1' OR  "Human T cell Leukaemia Virus 1") AND  ("Education" OR "Educational attainment"  OR "Educational status" OR "School" OR  'Schooling") |
| Income | ("HTLV-1" OR "HTLV-1 Infections" OR  "Human T-Lymphotropic Virus 1' OR  "'Human T cell Lymphotropic Virus 1' OR  "Human T cell Leukaemia Virus 1") AND  ("income" OR "Salary" OR "Salaries" OR  "Benefits" OR "Earning" OR "Minimum  wage" OR "Wage" OR "Socioeconomic  factors" OR "Social class" OR "Socioeconomic  status") |
| Employment | ("HTLV-1" OR "HTLV-1 Infections" OR  "Human T-Lymphotropic Virus 1" OR  "Human T cell Lymphotropic Virus 1' OR  "Human T cell Leukaemia Virus 1") AND  ("Employment" OR "Unemployment" OR  "Poverty") |
