# Supplementary material for: How do socioeconomic determinants of health affect the likelihood of living with HTLV-1 globally? A systematic review with meta-analysis
Source: Front Public Health. 2024 Jan 24;12:1298308. doi: 10.3389/fpubh.2024.1298308 (PMC10848500; doi:10.3389/fpubh.2024.1298308)
Supplement: Supplementary file 3 [file Table_3.docx]

| Study ID  **Table S3: Summary of characteristics of studies included in meta-analysis** | Country in which the study conducted | Study design | Population description | Inclusion criteria (if stated) | Exclusion criteria (if stated) | Total number of participants tested | How HTLV-1 was diagnosed | Determinant measured | Classification of determinant |
| --- | --- | --- | --- | --- | --- | --- | --- | --- | --- |
| Azarpazhooh 2012  AIDS RESEARCH AND HUMAN RETROVIRUSES  PMID: 22229796  Reference: 21 | Iran | Cross sectional study | Healthy residents selected by multistage cluster sampling from the Urban Health Cluster registry in Sabzevar, Iran | Healthy resident who is a member of the Urban Health Cluster registry in the 10 selected areas of Sabzevar, Iran |  | 1445 | PCR + ELISA | Education | Illiterate  Primary school  Secondary school  High school Academic |
| Barcellos 2006  Sexually transmitted diseases  PMID: **16505751**  **Reference: 22** | Brazil | Cross sectional study | Individuals testing for HIV in Rio Grande do Sul, Brazil. | >10 years of age  Had been screened for HIV between April-November 1996 | <10 years of age  Unconfirmed positive HIV test result Indeterminate WB for HTLV-1 | 2985 | ELISA + indirect immunofluorescence and Western Blot | Education | Incomplete high school  Completed high school |
| Blas 2013  PLoS One  PMID: **24040133**  Reference:23 | Peru | Cross sectional study | Shipibo-Konibo women (First nations people from Peruvian Amazon rainforest) between 15-39 years of age married/cohabitating for two or more years | Individuals between the ages of 15-17 who have been married/cohabiting for two or more years >18 years of age  Women belonging to the Shipibo-Konibo ethnic group | individuals <15 and >17 years old who are not married or cohabiting for two or more years, patients with indeterminate results in the WB for HTLV | 1253 | ELISA + WB | Education | <Primary education  >Primary education |
| Carneiro-Proietti 2012  AIDS Research and Human Retroviruses  PMID: **22324906**  Reference:24 | Brazil | Cross sectional study | Blood donors in Sao Paulo, Minas Gerais and Pernambuco, Brazil 2007 to 2009 | Blood donor at participating site |  | 281760 | EIA + WB | Education | High school  College  College+ |
| Castro Mendes 2020  Brazilian Journal of Microbiology  PMID: **31993990**  ^Reference: 25^ | Brazil | Cross sectional study | Pregnant women attending antenatal care in Maranhão, Brazil, February 2015 and May 2017. | Pregnant women  15-45 years of age |  | 713 | CMIA + WB and PCR | Education | <Full high school  >Full high school |
| Chen 1994  Journal of medical virology  PMID: **7911826** | Taiwan | Cross sectional study |  | Individuals involved with illicit drugs from  Yunlin  Penitentiary |  | 858 | ELISA + WB | Education  Employment | Education:  Elementary school  Junior high school  Senior high school  Employment:  Unemployed  Labourers  Salesmen  Prostitutes  Others |
| Costa 2018  Journal of Infection in Developing Countries  PMID: **31958329** | Brazil | Cross sectional study | Pregnant women attending the women reference attention centre in Ilheus city, Bahia, Brazil,July  2009–2010 | Pregnant women  Between 13-44 years of age |  | 726 | ELISA + WB + PCR | Education  Income | Education:  Elementary school or less (<8 years)  High school or more (>8 years)  Income (minimum wage):  <US $281.77  >US $281.77 |
| Courouble 2004  The West Indian Medical Journal  PMID: 11184174 | Guadeloupe, French West Indies | Cross sectional study | Blood donors | 18-65 years of age |  | 340 | Enzyme Immune Assay (EIA) + WB | Education  Income | Education:  Low level  High level  Income (monthly):  < US$1000  > US$1000 |
| Cunha 2007  Journal of medical virology  PMID: **17935167** | Mozambique | Cross sectional study | Replacement Blood donors | Donors attending the blood bank between April to July 2004 |  | 1577 | ELISA + WB with PCR for indeterminate WB samples | Education | No schooling experience Primary school |
| Dal Fabbro 2008  Revista da Sociedade Brasileira de Medicina Tropical  PMID: **18545834** | Brazil | Cross sectional study | Pregnant women | Pregnant women attending antenatal care program in Mato Grosso do Sul, Brazil, |  | 120,657 | Dried blood spot ELISA, followed by ELISA in serum, WB and PCR | Education | <3 years  4-7 years  8-11 years  >12 years |
| De Morais 2017  Revista do Instituto de Medicina Tropical de Sao Paulo  PMID: 29267588 | Brazil | Cross sectional study | Blood donors | Blood donors in Amazonas, Brazil, donating between August 2001-August 2003 |  | 87402 | ELISA + WB | Education | Elementary  High school Higher education |
| De Vita 2009  BMC Medical Research Methodology  PMID: **19284565** | United States of America | Cohort study | Blood donors | >18 years of age Tested either positive or negative for HTLV at the time of attempted donation HIV seronegative | <18 years of age | 1341 |  | Education  Income | Education:  High school or less  Some college  Bachelor's degree  Master's or professional degree  Income (annual):  <$10,000  $10,000-29,999  $30,000-49,999  $50,000-74,999  >$75,000 |
| Dourado 2003  Journal of Acquired Immune Deficiency Syndromes  PMID: **14657765** | Brazil | Cross sectional study | General population of Salvador, Brazil |  |  | 1385 | ELISA + WB+ PCR | Education  Income | Education:  <7 years  >7 years  Income (2.5 monthly minimum wage = US$125):  <2.5 minimum wage  >2.5 minimum wage |
| Emadi 2021  Transfusion and Apheresis Science  PMID: **33846093** | Iran | Retrospective descriptive study | Blood donors | Blood donors at Western Azerbaijan Regional Blood Transfusion Centre from May 2009 to May 2019 >18-56 years of age  Weight >50kg Haemoglobin level >12.5g/dL |  | 682,171 | ELISA + WB | Education | <12 years  >12 years |
| Falcão 2013  Journal of Medical Virology  PMID: **23852683** | Brazil | Transversal, analytical, population-based study between September 2008 and March 2010 | Members from two Brazilian Amazon communities, Pará | Individuals registered in the local family health program and have received primary healthcare in the local area  Individuals being screened for HTLV-1 |  | 657 | ELISA | Education  Income | Education:  < 4 years  > 4 years  Income:  <$305  >$305 |
| Ghaffari 2011  African Journal of Biotechnology  <http://dx.doi.org/10.5897/AJB11.177> | Iran | Cross sectional study | Hospital patients | Patients referred to Emam’s hospital laboratory in Sari between March 2009- March 2010 |  | 1200 | ELISA + WB | Education | Illiterate  Primary and secondary  University |
| Gotuzzo 1994  The Journal of Infectious Diseases  PMID: **8133088** | Peru | Cross sectional study | Female sex workers, Lima, Peru | Female sex workers attending an STI clinic Lima between October 1991 and April 1992 | Indeterminateresults on WB | 400 | ELISA + WB | Education | Elementary  High school  University/technical |
| Hananiya 2019  Journal of Immunoassay and Immunochemistry  PMID: **31339431** | Nigeria | Cross sectional study | Women attending post-natal clinics in Zaria in Kaduna State, Nigeria | Breastfeeding women attending post-natal care who consented, irrespective of age and education | Women not attending post-natal care | 190 | ELISA | Education  Employment | Education:  Informal Primary Secondary  Tertiary  Employment:  Unemployed  Self-employed  Civil servant  Others |
| Hedayati-Moghaddam 2011  Iranian Red Crescent Medical Journal  PMID: 22737506 | Iran | Cross sectional study | Patients being referred for HTLV-1 evaluation | All those who were referred to ACECR-Mashhad Clinical Lab in Neyshabour, North-eastern Iran for the evaluation of HTLV-1 infection |  | 511 | ELISA + WB | Education  Income  Employment | Education:  0-8 years  >9 years  Income:  <US $300  >US $300  Employment:  Employed/retired  Unemployed |
| Hedayati-Moghaddam 2015  Viruses-Basel  PMID: **26556363** | Iran | Case control study | Blood donors | First time blood donors Mashhad, Iran between September 2011 and August 2013 |  | 1022 | ELISA + WB | Education  Income | Education:  Illiterate  Primary school (1-5 years)  Secondary school (6-12 years)  Income (Million Rials):  <5  5-9.9  10.19.9 |
| Laguna-Torres 2005  Journal of Clinical Virology  PMID: **16461242** | Peru | Cross sectional study | Multi-transfused adult patients | >18 years of age  History of transfusion of a total of at least ten units of allogeneic blood or blood components (i.e., whole blood, plasma, red blood cells or platelets), received on at least two different occasions and/or with a history of at least three haemodialysis sessions  tudy subjects met at least one of five  criteria: (a) hemophlha or another coagulation disorder,  (b) hemodlalysls, (c) sickle-cell anemia or thalassemla,  (d) acute blood loss, or (e) oncologlc or hematological  disease February 2003 and September 200 | <18 years of age  Disseminated ecchymosis and/or bruises Any intravenous chemotherapy or active bleeding within 7 days prior to recruitment Platelet count <20000/ram 3 Haemoglobin <8 g/all Previous HBV or HCV refection prior to the first lifetime transfusion | 351 | ELISA + immunoblot | Education | Less than high school  High school  Technical  College |
| Maghsudlu 2015  Asian Journal of Transfusion Science  PMID: 26420946 | Iran | Cross sectional study | Blood donors | Blood donors donating at Sabzevar Blood Transfusion Centre between March 2009-April 2012 |  | 35,067 | ELISA + WB | Education | High school diploma or lower  Academic diploma |
| Mello 2014  Virology Journal  PMID: 24524416 | Brazil | Cross sectional study | Pregnant women | Pregnant women  Women treated at the antenatal units of the two largest hospitals located in Ilheus and Itabuna, Bahia, Brazil November 2008 and May 2010 |  | 2766 | ELISA + WB +PCR | Education  Income | Education:  Illiterate  Literate  Income (minimum wage):  <1  1-2  >2 |
| Moxoto 2007  Revista da Sociedade Brasileira de Medicina Tropical  PMID: **17486251** | Brazil | Other: Descriptive study | Women older than 14y attending hospital care at Bahia | >14 years of age | Pregnant women living with HIV | 130 | ELISA and WB, PCR for those WB Indeterminate | Education  Income | Education:  <8 years  >8 years  Income (minimum wage):  <1  1-3  3-5  >5 |
| Murphy 1996  International journal of epidemiology  PMID: **8921498** | Jamaica | Nested case-control study | Food service licence applicants |  |  | 426 | ELISA or WB (WB not done on all positive ELISAs) | Education  Income | Education:  Primary  Secondary or more  Weekly income in Jamaican $:  J$ 0-50  J$ 51-100  J$ 101-200  J$ 201+ |
| Murphy 1998  Journal of Acquired Immune Deficiency Syndromes and Human Retrovirology  PMID: **9525434** | United States of America | Cross sectional study | HTLV-I- or HTLV-II-positive and HTLV-negative blood donors | HTLV-1/2 positive or negative blood donors in the US |  | 1340 | ELISA + PCR | Education | <High school  >High school  Some college  >College |
| Murphy 1999  Archives of Internal Medicine  PMID: **10399901** | United States of America | Cohort study | Blood donors | HTLV-1/2 positive or negative blood donors in the US |  | 1340 |  | Income | Annual income (US$)  <30,000  30,000-50,000  >50,000 |
| Nunes 2017  PLoS One  PMID: 28158226 | Brazil | Cross sectional study | General population recruited through randomly sampling | Individuals living in Salvador, Brazil in one of 30 neighbourhoods |  | 3,451 | ELISA + WB | Education  Income | Education:  <7 years  >7 years  Income (monthly):  <2 minimum wages  >2 minimum wages |
| Olusanya 1990  Scandinavian Journal of Infectious Diseases  PMID: **1972590** | Nigeria | Cross sectional study | Individuals identifying as heterosexual recruited via a seroepidemiological project at a healthcare centre for business employees. | Healthy individuals between 15-42 years of age living in Ogun State, Nigeria  Individuals identifying as heterosexual |  | 385 | EIA + WB | Income | Low socioeconomic class  Middle and high socioeconomic class |
| Pegha Moukandja 2017  BMC Pregnancy and Childbirth  PMID: 28606185 | Gabon | Retrospective, cross-sectional study | Pregnant women | Pregnant women from May 2007 to July 2010 |  | 973 | Not explicitly mentioned for HTLV-1 | Employment | Worker  Student  Unemployed |
| Pessoni 2019  Hematology, Transfusion and Cell Therapy  PMID: **31409582** | Brazil | A retrospective analysis | Blood donors from Goiás, Brazil | First time and repeated blood donors |  | 137,209 | ELISA | Education | <12 years  >12 years |
| Rafatpanah 2011  Journal of Clinical Virology  PMID: **21840754** | Iran | Seroepidemiology study | Randomly selected members of the population in Mashhad, Iran (via multistage cluster sampling) | Randomly selected individuals living in Mashhad, Iran | Pilgrims and travellers to Mashhad, those who declined blood withdrawal | 1654 | ELISA + WB + PCR | Education  Income  Employment | Education:  Illiterate  Primary-secondary education (1-8 years)  High school (9-12 years)  Academic  Income (Million Rials per month e.g., approximately US $100):  <3  3-5  >5  Employment:  Employed  Unemployed |
| Reeves 1988  American Journal of Epidemiology  PMID: **2893539** | Panama | Cross sectional study | Individuals living in Panama City and Colon | >1 year of age  Consenting individuals | <1 year of age | 1451 | ELISA + competition with heterologous HTLV-1 antiserum | Income | Monthly household income:  Poor (<$75)  Low middle ($75-400)  Middle and upper ($500+) |
| Rouet 2002  Vox sanguinis  PMID: **11906668** | Guadeloupe, French West Indies | Case control study | Blood donors | HTLV-1 positive | Intravenous Drug Users (IDUs) Those having sex with commercial sex workers Those with sex partners who are IDUs  History of blood transfusion | 308 | EIA + WB | Income | Low socioeconomic status  Medium/high socioeconomic status |
| Schreiber 1997  Journal of Acquired Immune Deficiency Syndromes and Human Retrovirology  PMID: 9117460 | United States of America | Case control study | Blood donors |  |  | 1243 | EIA + PCR | Education | <High school graduate  Some college/technical  College graduate |
| Sequeira 2012  Revista da Sociedade Brasileira de Medicina Tropical  PMID: **22836660** | Brazil | Cross sectional study | Pregnant women | Pregnant women who tested positive for HTLV-1 with an anti-HTLV reagent test | Non-pregnant women | 13,382 | anti-HTLV ELISA test + WB | Education | Elementary  High school  Higher education |
| Silva 2018  Brazilian journal of epidemiology  PMID: **30328937** | Brazil | Prospective, Cross sectional study | Members of the public recruited by interviewing people passing through the Ver-o-Peso Complex and the Republic Square in the city of Belém, Pará, Brazil |  |  | 1059 | ELISA + PCR | Education  Income | Education:  0-11 years  >12 years  Income (minimum wage):  < 1  > 1 |
| Soares 2003  Revista De Saude Publica  PMID: **12937708** | Brazil | Cohort study | Blood donors, Hemominas blood centre, Minas Gerais, Brazil | Individuals who are considered eligible for blood donation according to pre-donation screening questionnaire and clinical examination  Aged 18-60 years  Good general health (no exposure to retrovirus risk factors e.g., use of illegal injecting drugs, unsafe sexual behaviour, tattoos)  Not having received blood or blood products transfusions in the last 10 years |  | 663 | EIA + WB | Education | <8 years  >8 years |
| SodreBarmpas 2019  PLoS neglected tropical diseases  PMID: **31181057** | Brazil | Cross sectional study | Pregnant women from Rio de Janeiro, Brazil, November 2012 to 2014 | Pregnant women | Women who lacked capacity to consent | 1628 | CMIA + WB | Education  Income | Education:  <10 years  >10 years  Income (monthly wage):  <2  >2 |
| Udeze 2018  Journal of Immunoassay and Immunochemistry  PMID: **30060717** | Nigeria | Cross sectional study | Pregnant women | 16-40 years of age  Pregnant women attending pre/antenatal care units in two public hospitals - General Hospital Ilorin and Civil Service Hospital Jan-May 2017 |  | 276 | ELISA | Education  Employment | Education:  <Secondary  >Secondary  Employment:  Trading  Schooling  Civil service  Artisanship  Housewife |
| Wang 1988  Cancer Research  PMID: **2900678** | Taiwan | Case control study | The total population of Taiwan Island, Penghu Islets, and Orchid Islet, with individuals  selected via multistage random sampling |  |  | 7278 | ELISA + WB | Education | Low education level  High education level |
| Yousefi 2020  Archives of Iranian Medicine  PMID: **33107308** | Iran | Cross sectional study | Individuals living in urban areas of Birjand city, Iran | 14-70 years of age |  | 3441 | ELISA | Education | Illiterate  Primary  Pre-high school  High school  University |
