# Supplementary material for: How do socioeconomic determinants of health affect the likelihood of living with HTLV-1 globally? A systematic review with meta-analysis
Source: Front Public Health. 2024 Jan 24;12:1298308. doi: 10.3389/fpubh.2024.1298308 (PMC10848500; doi:10.3389/fpubh.2024.1298308)

Figure S1. Forest plot comparing the level of secondary education and HTLV-1 prevalence, subdivided into the groups of general population, blood donors and pregnant women.


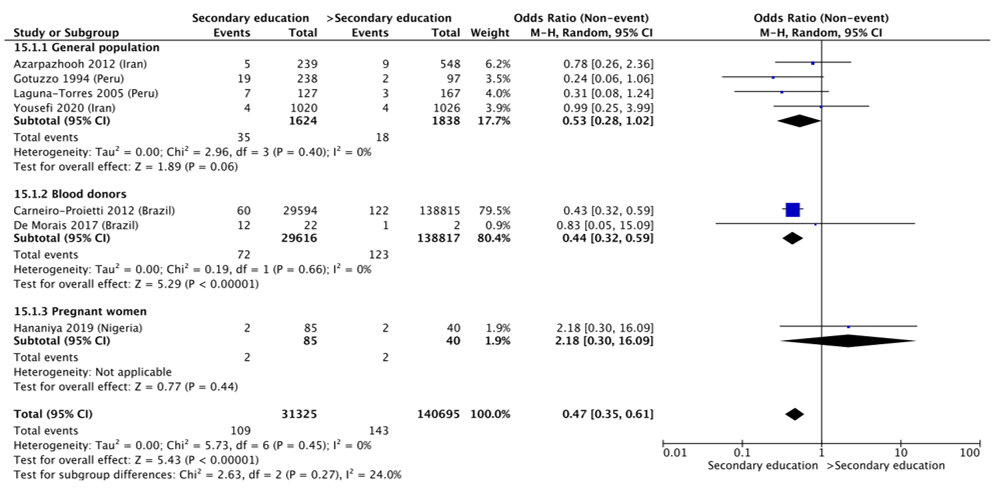

Supplement: Supplementary file 4 [file Data_Sheet_1.docx]
